# Second hand smoke exposure in workplace by job status and occupations

**DOI:** 10.1186/s40557-019-0282-z

**Published:** 2019-01-28

**Authors:** Hyunhee Park, Sung-il Cho, Changhun Lee

**Affiliations:** 1 Work Environment Research Bureau, Occupational Safety and Health Research Institute, 400, Jongga-ro, Jung-gu, Ulsan, Republic of Korea; 2 Department of Public Health Science, Graduate School of Public Health, and Institute of Health and Environment, Seoul National University, 1 Gwanak-ro, Gwanak-gu, Seoul, 08826 Republic of Korea

**Keywords:** Second hand smoke exposure, Job status, Occupations, Smoking ban

## Abstract

**Background:**

The objective of this study is to evaluate the risk of exposure to second hand smoke (SHS) during working hours by job status and occupation.

**Methods:**

Using the 4th Korean Working Conditions Survey (KWCS), 49,674 respondents who answered the question about SHS were studied. A chi-square test was carried out to determine whether there is a significant different in SHS exposure frequency by general and occupational characteristics and experience of discrimination at work and logistic regression analysis was carried out to identify the risk level of SHS exposure by variables.

**Results:**

In this study, we found that male workers in their 40s and 50s, workers employed in workplaces with fewer than 50 employees, daily workers, and people working outdoors had a higher rate of exposure to SHS than the others. The top five occupations with the highest SHS exposure were construction and mining-related occupations, metal core-makers-related trade occupations, wood and furniture, musical instrument, and signboard-related trade occupations, transport and machine-related trade occupations, transport and leisure services occupations. The least five exposed occupations were public and enterprise senior officers, legal and administrative professions, education professionals, and health, social welfare, and religion-related occupations.

**Conclusion:**

Tobacco smoke is a significant occupational hazard. Smoking ban policy in the workplace can be a very effective way to reduce the SHS exposure rate in the workplace and can be more effective if specifically designed by the job status and various occupations.

## Background

Second hand smoke (SHS), which is exposure to smoke from cigarette butts or smoke exhaled by smokers, is itself a Group 1 carcinogen for the human as classified by the International Agency for Research on Cancer (IARC). Exposure to SHS is known to be associated with respiratory and cardiovascular diseases as well as anxiety disorders, mental health, and psychological stress [1].

According to the National Health Statistics in Korea for 2015 [2], the current indoor SHS exposure rate of non-smokers at work was 26.8%, remarkably high compared with at home, which is 8.2%. From OECD (Organization for Economic Cooperation and Development) data [3], the smoking rate for Korean males aged more than 15 was 31.4% as of 2015, which was the third highest rank among OECD countries. The Korean government has implemented a policy of smoking bans in public places for many years, but that smoking policy only applies with a workplace more than 1000 m^2^ in total area [4]. For that reason, the workplace is still at a high rate of SHS exposure and could be the environment that can be improved further in SHS exposure reduction.

From 1999 to 2002 US NHANES (National Health and Nutrition Examination Survey) data, there have been dramatic reduction in the serum cotinine levels caused by successful smoking-free laws [5]. Although SHS exposure rates are declining, the workplace remains a significant source of SHS exposure [6, 7]. Working adults spend most of their time at workplace and for those non-smokers, the workplace may be the major source of provider to SHS exposure [8]. In the German study, More than 40% of non-smokers reported experiencing SHS at work [9]. Workplace smoking is an occupational health hazard and a smoking ban policy at the workplace is the best option to reduce SHS [10]. More than 50% of European countries enforce non-smoking regulations at work and the other European countries also partially restrict smoking at work. In the Netherlands, the comparison of SHS exposure rates before and after the implementation of the smoking ban policy in the workplace showed that the SHS exposure rate decreased from 70.7 to 51.9%. However, the rate of SHS exposure is still high in the Netherlands even after the smoking ban at work, because of a high-risk group for smoking such as males and low-educated workers [11].

To reinforce the appropriated non-smoking policy at workplace, it is important to identify priority group to implement such as vulnerable job status and occupations to SHS exposure. In this study, we evaluated the risk of exposure to tobacco smoke by others during working hours by job status, experience of discrimination at work and occupation using the data of the 4th Korean Working Conditions Survey (KWCS).

## Methods

### Study subjects

This study used data from the 4th Korean Working Conditions Survey (KWCS), which was conducted between June and September 2014 on employed workers by the Occupational Safety and Health Research Institute (OSHRI) affiliated under the Ministry of Employment and Labor. The KWCS selected individuals who satisfied criteria for the definition of “economically active population” conducted one-on-one interviews at their home by a professional interviewer. The total sample of 50,007 persons, 15 years or older participated in this survey. The data used in this study are from 49,674 respondents who answered the question “Are you exposed at work to tobacco smoke from other people?”

### Variables selected for analysis

The dependent variable was assessed to evaluate the risk of exposure to SHS by a question, “Are you exposed at work to tobacco smoke from other people?” Respondents answered on a seven-point scale of SHS exposure frequency; the choices were (in terms of all of the working time), “all”, “almost”, “3/4”, “half”, “1/4”, “almost never”, and “never”. For chi-square test, exposure to SHS in workplace was categorized into 3 group; “over 1/4 workhours” (all ~ 1/4), “almost never” and “never”. For logistic regression exposure to SHS in workplace was categorized into 2 group; “over 1/4 workhours” (all ~ 1/4) or “less than 1/4” (almost never and never). The rate of exposure to SHS for more than 1/4 of workhours (ESQ rate) was used to compare the SHS exposure by independent variables.

Independent variables included information on gender (“male” and “female”), age group ( “≤ 39”, “40–49”, “50–59” and “≥ 60”), job status (“self-employed without workers”, “self-employed with workers”, “wage workers (employees)”, “unpaid family worker” and “other workers”), type of wage worker (“permanent workers”, “temporary workers” and “daily workers”), wage provider (“workplace”, “a dispatcher” and “service provider”), Company size as number of workers in workplace ( “≤ 49”, “50–299” and “≥ 300”), type of workplace (“employer’s place of business”, “customer’s place of business”, “in the case of transportation as cars”, “outdoor (construction, field/etc)”, “my home” and “others”), job category (“manager”, “specialist”, “technician and associate export”, “office worker”, “service worker”, “sales worker”, “experts in agriculture and forestry fishing”, “functional person and related person”, “machine operator and assembly worker”, “laborer” and “soldier”), and night working days in a month ( “≤ 9”, “10–19” and “≥ 20”).

The item designed to evaluate the experience of discrimination at the workplace was used as a variables for the effect of exposure to SHS. The question is “During the past 12 months, did you experience to discrimination at your workplace related to age, race, nationality, gender, religion, disability, sexual orientation, academic group, region of origin, or employment status?” Respondents answered to the question about discrimination with ‘yes’ or ‘no’.

The occupational categories of respondents were classified according to the Korean Standard Classification of Occupation (KSCO by National Statistical Office) and classified into occupational groups (52 groups, classification code: 2 digits) and detailed occupation (415 groups, classification code: 4 digits).

### Statistical analysis

Data were analyzed using PASW version 18.0 (SPSS Inc., Chicago, IL, USA). Weight is applied when conducting statistical analysis based on the results of the “economically active population survey (EAPS)” in 2014 conducted by National Statistical Office. A chi-square test was carried out to determine whether there is a significant different between SHS exposure frequency by general and occupational characteristics and experience of discrimination at work and logistic regression analysis was carried out to identify the risk level of SHS exposure by variables.

## Results

### General characteristics of population

The sample characteristics are described in Table 1. Of the total 49,674 respondents, 57.8% were male and 36.9% were under the age of 39 which was the largest age group. The number of wage workers was 36,156 (72.8%) and number of respondents to the question about “wage worker type” and “wage provider” were 35,913 and 35,389 respectively. Of the respondents, 76.0% were permanent (regular) workers, 16.9% were temporary workers, and 7.1% were daily workers, respectively. Of the respondents, 94.7% were provided wage from workplace. By the size of the workplace, 79.7% were employed at a workplace with less than 50 employees. In the major categories of occupation, the office workers were the largest, followed by the service workers, the sales workers, and the simple laborers.

**Table 1:** General characteristics of the study subjects

Variables	n	Exposure to SHS in Workplace (Number (%))			*p*-value
		Over 1/4 workhours	Almost never	Never	
Gender					
Male	28,732 (57.8%)	4587 (16.0)	9163 (31.9)	14,982 (52.1)	*p* < 0.01
Female	20,942 (42.2%)	1441 (6.9)	5939 (28.4)	13,562 (64.8)	
Age(yrs)					
≤ 39	18,351 (36.9%)	1661 (9.1)	5341 (29.1)	11,349 (61.8)	*p* < 0.01
40–49	12,958 (26.1%)	1777 (13.7)	3929 (30.3)	7252 (56.0)	
50–59	11,348 (22.8%)	1698 (15.0)	3608 (31.8)	6042 (53.2)	
≥ 60	7017 (14.1%)	892 (12.7)	2224 (31.7)	3901 (55.6)	
Job status					
Self-employed without workers	8058 (16.2%)	915 (11.4)	2449 (30.4)	4694 (58.3)	*p* < 0.01
Self-employed with workers	3012 (6.1%)	522 (17.3)	913 (30.3)	1577 (52.4)	
Wage workers (employees)	36,156 (72.8%)	4344 (12.0)	10,956 (30.3)	20,856 (57.7)	
Unpaid family worker	2417 (4.9%)	244 (10.1)	767 (31.7)	1406 (58.2)	
Other workers	27 (0.1%)	3 (11.1)	14 (51.9)	10 (37.0)	
Type of wage workers					
Permanent workers	27,279 (76.0%)	2900 (10.6)	8459 (31.0)	15,920 (58.4)	*p* < 0.01
Temporary workers	6083 (16.9%)	722 (11.9)	1709 (28.1)	3652 (60.0)	
Daily workers	2551 (7.1%)	675 (26.5)	716 (28.1)	1160 (45.5)	
Wage_provider					
Work place	33,503 (94.7%)	3796 (11.3)	10,178 (30.4)	19,529 (58.3)	*p* < 0.01
A dispatcher	673 (1.9%)	104 (15.5)	202 (30.0)	367 (54.5)	
Service provider	1213 (3.4%)	345 (28.4)	340 (28.0)	528 (43.5)	
Company size (Number of workers in workplace)					
≤ 49	38,891 (79.7%)	4976 (12.8)	11,531 (29.6)	22,384 (57.6)	*p* < 0.01
50–299	6874 (14.1%)	670 (9.7)	2290 (33.3)	3914 (56.9)	
≥ 300	3011 (6.2%)	254 (8.4)	995 (33.0)	1762 (58.5)	
Type of workplace					
Employer’s place of business	37,873 (76.7%)	3949 (10.4)	11,486 (30.3)	22,438 (59.2)	*p* < 0.01
Customer’s place of business	4107 (8.3%)	694 (16.9)	1193 (29.0)	2220 (54.1)	
In the case of transportation such as cars	1388 (2.8%)	258 (18.6)	507 (36.5)	623 (44.9)	
Outdoor (construction site, field / etc.)	5304 (10.7%)	1039 (19.6)	1661 (31.3)	2604 (49.1)	
My house	533 (1.1%)	31 (5.8)	117 (22.0)	385 (72.2)	
Others	199 (0.4%)	16 (8.0)	52 (26.1)	131 (65.8)	
Job Category					
Manager	1354 (2.7%)	176 (13.0)	421 (31.1)	757 (55.9)	*p* < 0.01
Specialist	3759 (7.6%)	149 (4.0)	930 (24.7)	2680 (71.3)	
Technician and Associate Expert	2505 (5.0%)	279 (11.1)	797 (31.8)	1429 (57.0)	
Office worker	10,545 (21.2%)	746 (7.1)	3061 (29.0)	6738 (63.9)	
Service worker	7666 (15.4%)	874 (11.4)	2140 (27.9)	4652 (60.7)	
Salesperson	7532 (15.2%)	695 (9.2)	2115 (28.1)	4722 (62.7)	
Experts in agriculture and forestry fishing	2951 (5.9%)	213 (7.2)	1014 (34.4)	1724 (58.4)	
Functional Person and Related Person	4492 (9.0%)	1101 (24.5)	1572 (35.0)	1819 (40.5)	
Machine Operator and Assembly Worker	3349 (6.7%)	733 (21.9)	1281 (38.3)	1335 (39.9)	
Laborer	5416 (10.9%)	1049 (19.4)	1719 (31.7)	2648 (48.9)	
Soldier	83 (0.2%)	4 (4.8)	46 (55.4)	33 (39.8)	
Occasionally need to wear personal protective equipment					
Yes	12,071 (24.4%)	2709 (22.4)	4116 (34.1)	5246 (43.5)	*p* < 0.01
No	37,353 (75.6%)	3291 (8.8)	10,899 (29.2)	23,163 (62.0)	
Night working days in a month					
≤ 9	2896 (46.0%)	518(17.9)	874 (30.2)	1504 (51.9)	*p* < 0.01
10–19	2137 (34.0%)	392 (18.3)	763 (35.7)	982 (46.0)	
≥ 20	1256 (20.0%)	361 (28.7)	343 (27.3)	552 (43.9)	

From the chi-square test, the variables that showed significant differences in the exposure to SHS were gender, age, occupation status, wage provider, the size of the workplace, type of workplace, job category, whether to wear the personal protective equipment, and the number of night shifts. The rate of exposure to SHS for more than one quarter of working time (ESQ rate) was 16% for males and 6.9% for females. By age, the ESQ rate was highest in the 50s and by employment status, the ESQ rate was highest for self - employed with workers. Among the wage workers, the ESQ rate was highest for the daily workers, and in terms of the wage-payment method, the ESQ rate was highest for workers who were paid by service companies. In terms of the number of employees at workplaces, the ESQ rate was highest for the companies with less than 50 employees and by types of workplace, the ESQ rate was highest in outdoor work-places, such as construction sites and farms. By types of occupation, the ESQ rate was highest in the functional and related functional staff. The rate of SHS exposure was higher for workers wearing protective gear than for workers not wearing protective gear. A greater- number of night shifts also increased SHS exposure (Table 1).

### SHS exposure by job status

According to the logistic regression analysis (Table 2), the risk of SHS exposure of males was 4.107 times (95% CI: 3.461 ~ 4.874) higher than that of females. By age groups, exposure for those in their forties was 1.551 times (95% CI: 1.348 ~ 1.785) and for those in their fifties were 1.529 times (95% CI: 1.319 ~ 1.772) more than for those under 39 years of age. In terms of the number of workers in the workplace, the risk of exposure to SHS in companies with less than 50 workers was 2.114 times (95% CI: 1.721 ~ 2.595) higher than that in companies with more than 300 workers and that of daily workers was 1.318 times (95% CI: 1.102 ~ 1.575) higher than that of regular workers. The risk of exposure to SHS for workers receiving wages from the service provider was 1.736 times higher than that for workers receiving wages at work. The risk of exposure to SHS for outdoor workers was 1.668 times higher than for those working at the employer’s place of business.

**Table 2:** Multiple logistic analysis of factors affecting secondhand smoke exposure in workplace

Dependent variables	Adjusted OR (Odds Ratio)	95% CI (Confidence Interval)
Sex (reference: Female)		
Male	4.107**	3.461 ~ 4.874
Age (reference: ≤39)		
40–49	1.551**	1.348 ~ 1.785
50–59	1.529**	1.319 ~ 1.772
≥ 60	1.116	0.916 ~ 1.360
Number of workers in workplace (reference: ≥ 300)		
≤ 49	2.114**	1.721 ~ 2.595
50–300	1.545**	1.232 ~ 1.937
Status (reference: Permanent workers)		
Temporary workers	1.130	0.957 ~ 1.334
Daily workers	1.318**	1.102 ~ 1.575
Occasionally need to wear personal protective equipment (reference No)		
Yes	1.132	0.942 ~ 1.359
Wage_Provider (reference: Work place (last week’s work place))		
A dispatcher	1.140	0.814 ~ 1.596
Service provider	1.736**	1.401 ~ 2.149
Type of Workplace (reference: Employer’s place of business		
Customer’s place of business	1.220*	1.014 ~ 1.466
In the case of transportation such as cars	.696	0.466 ~ 1.040
Outdoor (construction site, field / etc.)	1.668**	1.423 ~ 1.956
My house	2.200	0.186 ~ 25.995

Adjusted by Sex, Age, Number of workers in workplace, Work status, need of personal protective equipment, Wage provider and Types of workplace

* *p* < 0.05 ** *p* < 0.01

### SHS exposure by experience of discrimination at work

Examining the degree of exposure to SHS in terms of experience of discrimination in the last 12 months in the workplace, showed that workers who have experienced discrimination at work because of age (adjusted OR 1.637, 95% CI 1.468~1.825), race (adjusted OR 1.850, 95% CI 1.459~2.347), nationality (adjusted OR 2.699, 95% CI 2.161~3.371), sex (adjusted OR 1.969, 95% CI 1.623~2.389), disability (adjusted OR 2.758, 95% CI 2.069~3.676), sexual orientation (adjusted OR 1.801, 95% CI 1.231~2.633), academic group (adjusted OR 1.271, 95% CI 1.119~1.443), place of origin (adjusted OR 1.657, 95% CI 1.384~1.984), or employment status (adjusted OR 2.010, 95% CI 1.770~2.283) were more exposed to SHS than the counterparts who are not experienced discrimination (Tables 3 and 4).

**Table 3:** Secondhand smoke exposure affecting by discrimination experience

Variables	n	Exposure to SHS in Workplace (Number (%))			*p*-value
		Over 1/4 workhours	Almost never	Never	
Age discrimination					
Yes	2473 (5.0%)	438 (17.7)	739 (29.9)	1296 (52.4)	*p* < 0.01
No	46,996 (95.0%)	5546 (11.8)	14,291 (30.4)	27,159 (57.8)	
Race discrimination					
Yes	442 (0.9%)	89 (20.1)	131 (29.6)	222 (50.2)	*p* < 0.01
No	49,046 (99.1%)	5913 (12.1)	14,883 (30.3)	28,250 (57.6)	
Nationality discrimination					
Yes	417 (0.8%)	113 (27.1)	122 (29.3)	182 (43.6)	*p* < 0.01
No	49,081 (99.2%)	5888 (12.0)	14,899 (30.4)	28,294 (57.6)	
Sex discrimination					
Yes	802 (1.6%)	132 (16.5)	240 (29.9)	430 (53.6)	*p* < 0.01
No	48,698 (98.4%)	5859 (12.0)	14,803 (30.4)	28,036 (57.6)	
Religion discrimination					
Yes	143 (0.3%)	13 (9.1)	58 (40.6)	72 (50.3)	*p* < 0.01
No	49,353 (99.7%)	5984 (12.1)	14,982 (30.4)	28,387 (57.5)	
Disability discrimination					
Yes	244 (0.5%)	68 (27.9)	77 (31.6)	99 (40.6)	*p* < 0.01
No	49,197 (99.5%)	5924 (12.0)	14,930 (30.3)	28,343 (57.6)	
Sexual orientation discrimination					
Yes	188 (0.4%)	34 (18.1)	70 (37.2)	84 (44.7)	*p* < 0.01
No	49,212 (99.6%)	5960 (12.1)	14,925 (30.3)	28,327 (57.6)	
Academic group discrimination					
Yes	2113 (4.3%)	299 (14.2)	596 (28.2)	1218 (57.6)	*p* < 0.01
No	47,262 (95.7%)	5677 (12.0)	14,375 (30.4)	27,210 (57.6)	
Region of origin discrimination					
Yes	820 (1.7%)	154 (18.8)	261 (31.8)	405 (49.4)	*p* < 0.01
No	48,610 (98.3%)	5831 (12.0)	14,743 (30.3)	28,036 (57.7)	
Employment status discrimination					
Yes	1593 (3.2%)	327 (20.5)	460 (28.9)	806 (50.6)	*p* < 0.01
No	47,782 (96.8%)	5659 (11.8)	14,531 (30.4)	27,592 (57.7)

**Table 4:** Multiple logistic analysis of factors affecting second hand smoke exposure of discrimination experience

Dependent variables	OR(Odds Ratio, 95% CI)	
	Crude OR	Adjusted OR
Age discrimination (reference No)		
Yes	1.607**(1.444~1.789)	1.637**(1.468~1.825)
Race discrimination (reference No)		
Yes	1.848**(1.463~2.335)	1.850**(1.459~2.347)
Nationality discrimination (reference No)		
Yes	2.719**(2.186~3.380)	2.699**(2.161~3.371)
Sex discrimination (reference No)		
Yes	1.444**(1.196~1.744)	1.969**(1.623~2.389)
Religion discrimination (reference No)		
Yes	0.748(0.426~1.315)	0.837(0.473~1.480)
Disability discrimination (reference No)		
Yes	2.820**(2.128~3.736)	2.758**(2.069~3.676)
Sexual orientation discrimination (reference No)		
Yes	1.594*(1.098~2.315)	1.801*(1.231~2.633)
Academic group discrimination (reference No)		
Yes	1.207*(1.065~1.368)	1.271**(1.119~1.443)
Region of origin discrimination (reference No)		
Yes	1.697**(1.421~2.027)	1.657**(1.384~1.984)
Employment status discrimination (reference No)		
Yes	1.922**(1.697~2.178)	2.010**(1.770~2.283)

Adjusted by age and sex

* *p* < 0.05 ** *p* < 0.01

### SHS exposure by occupations

Occupational groups (classification code: 2 digits) by KSCO were analyzed and classified into 52 groups. Construction and mining-related occupations (49.5%), metal coremakers-related trade occupations (33.3%), and transport and machine-related trade occupations (31.4%) were the highest exposure groups for SHS at the workplace. Public and enterprise senior, legal and administration professional occupations (0.9%), education professional and related occupations (1.7%), and health, social welfare, and religion-related occupations (1.7%) were the lowest in exposure to SHS at the workplace (Table 5).

**Table 5:** Exposure to second hand smoke in workplace by occupation group (Top20)

Occupation Group (Code)^a^	n	The rate of exposure SHS in workplace over 1/4 working hours (Number (%))
Construction and Mining Related Elementary Occupations(91)	671	332 (49.5)
Construction and Mining Related Trade Occupations(77)	1056	433 (41)
Metal Coremakers Related Trade Occupations(74)	393	131 (33.3)
Wood and Furniture, Musical Instrument and Signboard Related Trade Occupations(73)	114	37 (32.5)
Transport and Machine Related Trade Occupations(75)	759	238 (31.4)
Transport and Leisure Services Occupations(43)	328	101 (30.8)
Wood, Printing and Other Machine Operating Occupations(89)	290	87 (30)
Other Technical Occupations(79)	259	75 (29)
Skilled Forestry Occupations(62)	12	3 (25)
Skilled Fishery Occupations(63)	73	17 (23.3)
Construction, Electricity and Production Related Managers(14)	181	41 (22.7)
Driving and Transport Related Occupations(87)	2503	539 (21.5)
Metal and Nonmetal Related Operator Occupations(84)	233	47 (20.2)
Machine Production and Related Machine Operators(85)	1069	214 (20)
Electric and Electronic Related Trade Occupations(76)	458	89 (19.4)
Chemical Related Machine Operating Occupations(83)	283	54 (19.1)
Video and Telecommunications Equipment Related Occupations(78)	108	20 (18.5)
Police, Fire Fight and Security Related Service Occupations(41)	401	70 (17.5)
Transport Related Elementary Occupations(92)	713	118 (16.5)
Clean and Guard Related Elementary Occupations(94)	2120	341 (16.1)

^a^Korean Standard Classification of Occupation code (2 digit code)

Specific jobs (classification code: 4 digits) by KSCO were analyzed and classified into 415 jobs. Of these, 151 jobs with 50 or more respondents were analyzed. The top 20 jobs for exposure to SHS for more than a quarter of working time are shown in Table 6. Concrete-reinforcing iron workers (53.7%), plasters (52%), construction and mining elementary workers (49.5%), construction plumbers (48.9%), and entertainment facilities workers (47.9%) were the highest SHS exposure jobs. Among the top 50 jobs, construction jobs accounted for about a dozen (Table 6).

**Table 6:** Exposure to second hand smoke in workplace by specified jobs (Top 20)

Specified job (Code)^a^	n	The rate of exposure SHS in workplace over 1/4 working hours (Number (%))
Concrete Reinforcing Iron Workers(7721)	67	36 (53.7)
Plasters(7731)	102	53 (52)
Construction and Mining Elementary Workers(910)	671	332 (49.5)
Construction Plumbers(7921)	88	43 (48.9)
Entertainment Facilities Workers(4323)	192	92 (47.9)
Construction Painters(7736)	94	41 (43.6)
Floor Installers(7734)	61	26 (42.6)
Automobile Mechanics(751)	418	170 (40.7)
Window Chassis Assembers and Installers(7737)	87	35 (40.2)
Construction Carpenters(7724)	269	102 (37.9)
Furniture Makers and Repairers(7302)	66	25 (37.9)
Other Construction Finishing Related Technical Workers(7739)	99	37 (37.4)
Printing Machine Operators(8921)	170	63 (37.1)
Street Stall Salespersons and Vendors(5305)	79	29 (36.7)
Welders(743)	335	122 (36.4)
Cutters(7212)	68	23 (33.8)
Interior Electricians(7622)	172	56 (32.6)
Construction and Mining Related Managers(1411)	109	33 (30.3)
Machine Tool Operators(851)	344	100 (29.1)
Handling Equipment Operators(874)	239	69 (28.9)

^a^Korean Standard Classification of Occupation code (4 digit code)

## Discussion

### By job status

In this study, we found that male workers in their 40s and 50s, workers employed in workplaces with fewer than 50 employees, daily workers, temporary workers, and people working at the customer’s premises or working outdoors had a higher risk of exposure to SHS than the others. The Dutch study reported that workers who were male and low-educated were more likely to be exposed to SHS [8]. The German study reported the aspect of higher SHS exposure in younger age group, but this is dependent on the place of exposure, and exceptionally at workplace, 30–44 years had highest SHS exposure differ by the others such as home, bars, or the house of friend [9]. It is known that blue-collar workers and service workers are more likely to expose the higher rate of SHS occupationally than white-collar workers. These are serious concern because blue collar workers have exposed more often to chemical and dust and SHS related health problems can be synergistically effect with those hazards [10].

Also, we found workers who had experienced discrimination at workplace based on age, race, nationality, gender, disability, academic group, place of origin, or type of employment were more likely to experience a higher rate of exposure to tobacco smoke than were their counterparts who had not experienced discrimination. A recent study reported that exposure to discrimination based on age, academic group or employment status put the workers at a high risk of having a poor well-being [12]. Another study also reported an association between SHS and psychological well-being and emphasized the importance of reducing SHS exposure at the workplace [13]. From these studies, exposure to discrimination and SHS are both significantly more likely to induce a poor well-being than counterparts who were not exposed to discrimination and SHS.

### By occupations

The top ten occupations with the highest SHS exposure were construction and mining-related occupations, construction and mining-related trade occupations, metal coremakers-related trade occupations, wood and furniture, musical instrument, and signboard-related trade occupations, transport and machine-related trade occupations, transport and leisure services occupations, wood, printing and other machine-operating occupations, other technical occupations, and skilled forestry occupations, skilled fishery occupations. The least exposed occupations were public and enterprise senior officers, legal and administrative professions, education professionals, and health, social welfare, and religion-related occupations. Wortley et al. (2002) [8] compared the levels of serum cotinine in nonsmokers to assess the risk of SHS exposure based on NHANES III (1988–1994) and found that among 40 occupational groups, the geometric mean of serum cotinine was highest in the waiter and waitress group (0.47 ng/mL), among seven job categories, the geometric mean of serum cotinine was highest in the device operation, producer, and laborer (0.22 ng/mL). The research data related SHS exposure by occupation were rare to find, instead we can refer to the smoking rate by occupations. Based on the NHANES(National Health and Nutrition Examination Survey) III in the United States, 1988–1994, smoking rates by occupation showed that material-moving occupations, construction laborers, and vehicle mechanics and repairers had the highest smoking rate, whereas teachers and sales representatives reported that the rate of smoking was low [14]. Smith and Leggat (2007) [15] reported that by job category, smoking was most common among laborers and least common among professionals, managers, or administrators. Occupations with high smoking rates were very similar to occupations with high SHS rates in our study. Tobacco smoke represents an occupational hazard and a smoke-free environment is an essential component of a healthy and safe. Smith and Leggat [15] reported that smoking rates were higher among unemployed persons in many European countries like France, Italy, and Sweden, the United States and Australia, whereas in Japan, people who were currently employed actually had the higher smoking rates. These results can be affected by different working condition including job status and occupation of each country. Therefore it is necessary to analyze the smoking and SHS exposure rates in job status and occupation for each country.

The strength of this study is to analyze the SHS exposure by job status and occupations and identify the priority group to implement smoking ban policy and confirm the workplace smoking is an occupational health hazard and a smoking ban policy at the workplace is the best option to reduce SHS. But, the limitation of study is that in 4th KWCS data, participant’s smoking status was not surveyed and we have not been able to identify how much SHS exposure is affected by whether or not participants are smokers.

## Conclusions

Tobacco smoke is a significant occupational hazard. Smoking cessation ban in the workplace can be a very effective way to reduce the SHS rate in the workplace and can be more effective if specifically designed for the various occupations and working styles in each country. Particularly in South Korea, workers in their 40s and 50s, workers employed in workplaces with less than 50 employees, daily workers and temporary workers, workers who work outside, and construction workers are priority target for non-smoking regulations at work.
